# Clinicopathological spectrum of ovarian sex cord-stromal tumors; 20 years’ retrospective study in a developing country

**DOI:** 10.1186/1757-2215-6-87

**Published:** 2013-12-05

**Authors:** Saroona Haroon, Aleena Zia, Romana Idrees, Aisha Memon, Saira Fatima, Naila Kayani

**Affiliations:** 1 Section of histopathology, Department of Pathology & Microbiology, Aga Khan University Hospital, P.O. Box 3500 Karachi, Pakistan; 2 Medical student, The Aga Khan University, Karachi, Pakistan

**Keywords:** Sex cord stromal tumour, Granulosa cell tumour, Ovarian neoplasm

## Abstract

**Background:**

Ovarian sex cord stromal tumors are rare neoplasms as compared to epithelial tumors. No large study has been done in Pakistan to find out the frequencies of various sex cord stromal tumors and their clinicopathological behavior in our region. The purpose of our study was to determine the various histological patterns and clinical features of ovarian sex cord stromal tumors along with follow-up in our set-up.

**Methods:**

It is a retrospective observational study. The study was conducted in section of Histopathology, Aga Khan University Hospital, Karachi, Pakistan. All reported cases of sex cord stromal tumors of ovary during 1992 to 2012 were retrieved. The retrieved slides were reviewed and patient demographics, clinical and pathological features were noted on proforma. SPSS Statistics Version 19 was used for all analyses. Data is expressed as absolute values and percentage or as mean ± standard deviation (SD).

**Results:**

A total of 480 cases of sex cord stromal tumors were retrieved. The median age was 45 years. Bilaterality was observed in 4 cases. Of the different subtypes of sex-cord stromal tumors, most common was adult granulosa cell tumor 211(43.9%). 24 Juvenile granulosa cell tumors were retrieved (5%). Other types were fibromas 98 (20.4%) fibrothecomas 47(9.8%), thecomas 26(5.4%), sertoli-leydig cell tumors 34(7%), sclerosing stromal tumors 26 (5.4%), steroid cell tumors (10) and 4 cases of sex cord tumor with annular tubules. Of various immunohistochemical stains applied, Inhibin was frequently positive in all subtypes and focal cytokeratins were also seen commonly. Follow up information was available in 305 cases and out of these only 16 (5%) developed recurrence or metastasis.

**Conclusions:**

Sex cord stromal tumors are uncommon ovarian tumors in Pakistani population, with wide age range and diverse histological types having good prognosis. Immunohistochemical markers overlap with epithelial tumors so there is need to distinguish these two.

## Background

Sex-cord stromal tumors (SCST) are subtype of ovarian neoplasms that are relatively infrequent. These account for only about 7% of all primary ovarian tumors. The sex cord stromal tumors are most of the time low grade and present generally in younger patients than ovarian epithelial malignancies [1]. They encompass a heterogeneous group of neoplasms containing variety of cells which are derived from gonadal sex cords or stromal cells [2]. Because of the smaller size, lower grade of malignancy and the rarity of these tumors, they are often diagnosed by pathology following surgery [3].

The morphology of these tumors varies and these can simulate epithelial ovarian neoplasms or mesenchymal tumors histologically to an extent of misdiagnosis [4]. Immunohistochemical staining may be useful for establishing the diagnosis in problematic cases due to varied appearance and rarity [3,5]. Steroidenic factor 1 (SF 1) and FOXL2, although not available in our set-up are useful marker studied in recent times and is positive in lesions of sex cord-stromal differentiation. Although some of these tumors are Cytokeratin (CK) AE1/AE3 positive, epithelial membrane antigen (EMA) negativity may be useful for the differential diagnosis with epithelial ovarian tumors [6].

Furthermore these tumors can show somewhat fascinating behavior profile with the fluctuating clinical presentations of precocious puberty to menorrhagia to postmenopausal bleeding [7].

Primary treatment is surgery, which is conservative for stage-1 tumors. Treatment for advanced or recurrent disease includes primary or adjuvant chemotherapy. Majority of these tumors are of low malignant potential and are associated with favorable prognosis [8].

We performed the research to analyze the frequencies and clinicopathological spectrum along with follow up of ovarian sex cord stromal tumors in our center, which is one of the largest referral centers for histopathology specimens in Pakistan.

## Methods

We performed a retrospective analysis of all patients diagnosed with ovarian sex cord stromal tumors from January 1, 1992 to December 31, 2012, whose specimens were received at Histopathology section, Department of Pathology and Microbiology, Aga Khan University Hospital, Karachi. Patients were identified from a prospectively maintained departmental database, Integrated Lab. Management System, with keywords search. As this is an observational study and the confidentiality was thoroughly maintained, ethical review committee’s approval was not required, which is mandatory in experimental research according to Helniski’s declaration.

Ovarian sex cord stromal tumors were diagnosed according to defined morphology and immunohistochemistry. More specifically, granulosa cell tumors (both juvenile and adult types), fibromas, thecomas, sclerosing stromal tumors, sex cord tumor with annular tubules, Sertoli-leydig cell tumors, steroid cell tumors or mixture of these were included in this study. For patients that presented with recurrence, slides from the initial specimen (if available) were reviewed to confirm the diagnosis. Following high laboratorial quality control systems, Five-micrometer thick sections were used for the immunohistochemistry studies. The tissue samples were processed by conventional methods, including overnight fixation in 10% formalin and subsequent embedding in paraffin.

The most representative blocks were used and stained with haematoxylin and eosin. Immunohistochemical assessment was carried out using the avidin–biotin immunoperoxidase technique. The primary antibodies used in the study are outlined in Table 1. Relevant cytoplasmic and/or membranous staining by antibodies was taken as positive according to established criteria.

**Table 1:** Common antibodies used in the study

**Antibody**	**Clone**	**Species**	**Antibody source**	**Dilution**
Inhibin	R1	Monoclonal Mouse Anti-Human	Dako Denmark	1:50
Actin (Smooth Muscle)	1A4	Monoclonal Mouse Anti-Human	Dako Denmark	1:250
Vimentin	Vim 3B4	Monoclonal Mouse Anti-Human	Dako Denmark	1:100
CD 99, mic-2	12E7	Monoclonal Mouse Anti-Human	Dako Denmark	Pre-diluted
Cytokeratin	AE1/AE3	Monoclonal Mouse Anti-Human	Dako Denmark	1:50
Calretinin	DAK-Calret 1	Monoclonal Mouse Anti-Human	Dako Denmark	1:100
Epithelial Membrane Antigen (EMA)	E29	Monoclonal Mouse Anti-Human	Dako Denmark	1:50
Cytokeratin 7	OV-TL 12/30	Monoclonal Mouse Anti-Human	Dako Denmark	1:100
CD117, c-kit	-	Polyclonal Rabbit Anti-Human	Dako Denmark	1:100
Desmin	D33	Monoclonal Mouse Anti-Human	Dako Denmark	1:150
Cytokeratin	CAM 5.2	Monoclonal Mouse Anti-Human	Becton, Dickinson and Company, NJ	Pre-diluted

We excluded patients where differential diagnosis was given, due to non-convincing histology and/or suboptimal immunostaining. Electronic medical records were reviewed for age at diagnosis, surgical procedures performed, site of tumor and type of tumor. Surgical stage was determined from pathologic records at the time of diagnosis, when available. Complete staging was defined as at least a unilateral salpingo-oophorectomy, pelvic washings, peritoneal biopsies, omental sampling, and pelvic and para-aortic lymph nodal sampling. Surgical stage based on the International Federation of Gynecology and Obstetrics (FIGO) 1988 criteria was compared to the recent FIGO 2009 staging criteria modifications. Staging procedures were predominantly done for clinically suspicious and per-operatively malignant cases. Few patients whose surgery was performed in our own centre or in vicinity had intra-operative frozen section.

For the present study, all relevant data were retrieved from our database into a separate anonymous database. In this separate database, patient identity was protected by a study-specific, unique patient code, which was known only to two of the authors (SH and AZ).

This is a single center retrospective case series. SPSS Statistics Version 19 was used for all analyses. Data are expressed as absolute values and percentage or as mean ± standard deviation (SD).

## Results

A total of 480 patients with ovarian sex cord stromal tumors were included with a median age of 45 years. The age ranged from 1 to 92 years with mean ± SD was 44.3 ± 16.8 years. The right ovary was involved in 199 cases (55.1%) and left in 162 cases (44.9%), Bilaterality was observed in 4 cases. In the rest of cases, information regarding laterality was missing. Abdominal lump with pain (67%) and abdominal distension (54%) were the most common symptoms (Table 2).

**Table 2:** Age distribution and frequencies of various clinical signs and symptoms

**Age of patient (years)**	**Number of patients**
**<20**	34
**≥20-40**	145
**≥40-60**	186
**≥60-80**	108
**≥80**	7
**Presenting complaints**	
**Asymptomatic**	87
**Lump with pain abdomen**	218
**Irregular bleeding per-vaginum**	136
**Postmenopausal bleeding**	68
**Secondary amenorrhea**	10
**Precocious puberty**	7
**Virilizing symptoms**	39
**Overlapping symptoms**	81

### Granulosa cell tumor, adult type

**Figure 1:**
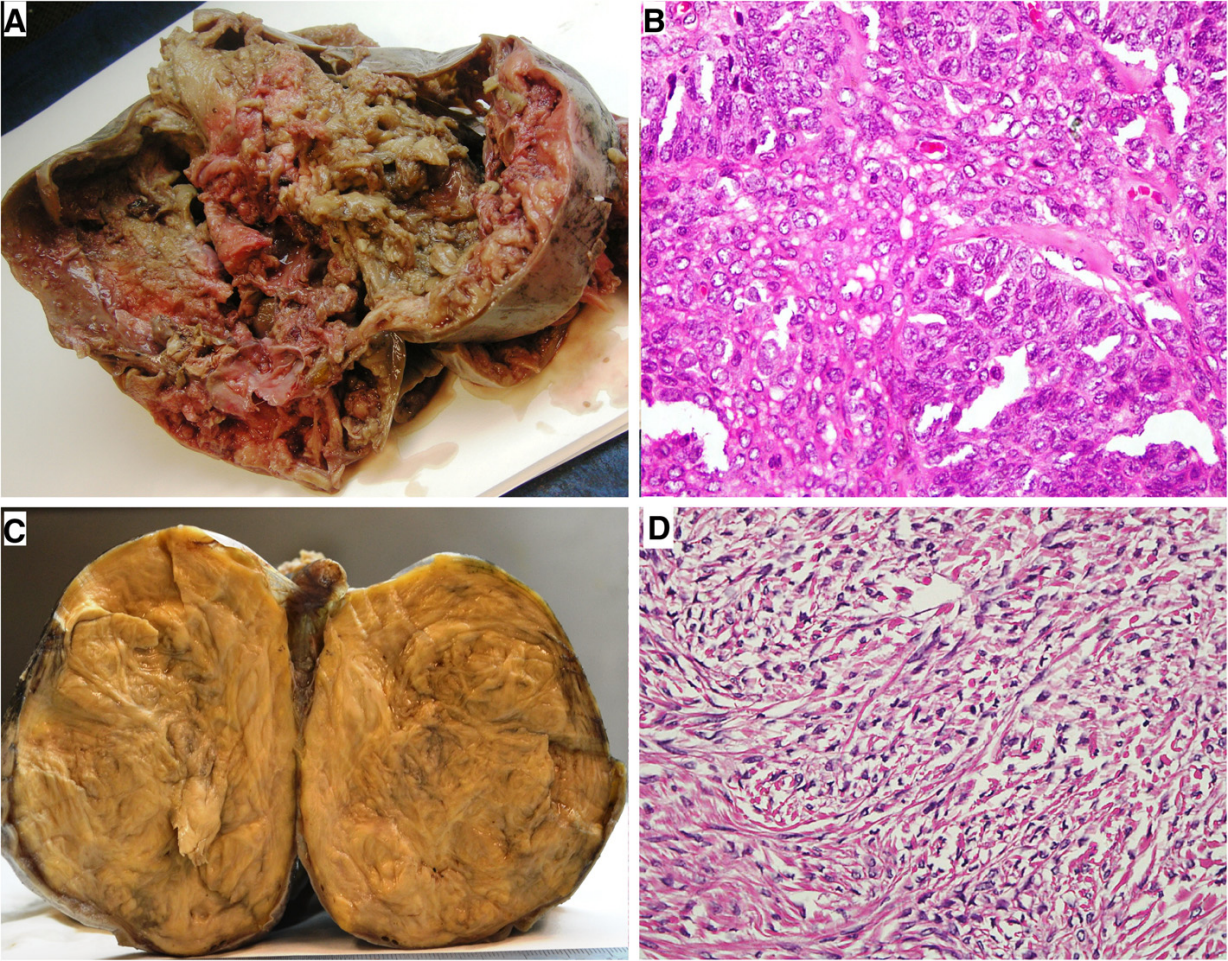
**Gross and microscopic features of two most frequent SCSTs; Adult granulosa cell tumor and Fibroma. A)** Gross appearance of Adult granulosa cell tumor. **B)** Microscopic picture shows coffee bean nuclei of the tumor cells present in form of sheets. (Hemotoxylin and Eosin, 40×). **C)** Gross picture of Fibroma. **D)** Microscopy shows bland nuclei with scant cytoplasm. (Hemotoxylin and Eosin, 40×).

Of the different types of sex-cord stromal tumors adult granulosa cell tumor (AGCT) was most common subtype amounting to total number of 211(43.9%). Age range was 19 to 90 years with median age of 48 years. Right sided ovary was slightly more involved (81 cases) than left sided ovary (71 cases). Most of the tumors had solid and partially cystic hemorrhagic surface with tumor cells having typical coffee bean nuclei on light microscopy (Figure 1).

Detailed immunohistochemical analysis was done in 163 cases, which showed all of granulosa cell tumors positive for Inhibin, majority i.e. approximately 90% were positive for calretinin (n = 147) , 68% were positive for CD99 (n = 111), and a large number approximately 60% were also weak focal positive for Cytokeratins (n = 98).

Twenty four granulosa cell tumors were associated with simple endometrial hyperplasia without atypia. Three of the granulosa cell tumors were associated with endometrial carcinoma and one with endometrial hyperplasia with atypia and one with endometrial polyp.

Total 13 cases showed metastasis or the tissue received was from sites adjacent to ovary. Out of which ten involved mesentery/omentum, one infiltrated small intestine and one infiltrated large intestine. One even reached the anterior abdominal wall. One cases surprisingly had synchronous squamous cell carcinoma of cervix.

The various types of specimen reflecting the nature of surgery performed in each kind sex cord stromal tumor are listed in Table 3. Complete follow-up data of only 86 patients was available, with late recurrence in 5 cases (at 18, 23, 37, 41 and 52 months after initial diagnosis). Metastasis was observed in 4 patients. 2 patients died of AGCT (11 and 18 months respectively after initial diagnosis) and 2 patients died of some other illness. Rests of patients were free of disease till the time of last follow up.

**Table 3:** Frequencies of various types of sex cord stromal tumors, median age and mean size

**Type of tumor N (percentage)**	**Median age (in years)**	**Mean size (in cms)**	**Types of specimen received**			
			**TAH-BSO**	**USO**	**BSO**	**TAH-USO**
Adult granulosa cell tumor 211 (43.9%)	48	10.8	121	23	17	32
Juvenile granulosa cell tumor 24 (5%)	19	15	7	5	3	5
Fibrothecoma 47 (9.8%)	50	10.9	15	21	4	3
Fibroma 98 (20.4%)	40	8.7	32	16	6	9
Thecoma 26 (5.4%)	52	8.5	6	8	2	2
Sertoli Leydig cell tumor 34 (7%)	30	15.4	10	6	2	1
Steroid cell tumor 10 (2%)	44	6.3				
Sclerosing stromal tumor 26 (5.4%)	22.5	9.3	7	4	1	6
Sex cord tumor with annular tubules 4 (0.8%)	32.5	9				

Abbreviations:

*TAH-BSO* Total abdominal hysterectomy and bilateral salpingo-oophorectomy.

*USO* Unilateral salpingo-oophorectomy.

*BSO* Bilateral salpingo-oophorectomy.

### Granulosa cell tumor, juvenile type

Total 24 juvenile granulosa cell tumors (JGCT) were diagnosed during this 20 year period. Immunohistochemical analysis done in 20 cases showed negative expression of CKs. Follow up of only 11 cases was available, and out of these 2 cases had recurrence (after 8 and 17 months of initial diagnosis) and one died of disease (after 7 months).

### Fibromas

Total 98 cases of fibromas were found. Age range was 12 to 79 years and mean was 42.2 years. Cellular fibromas had mean size larger (14.5 cm) than those of fibromas (8.7 cm). The gross appearance of fibromas showed whorled tan white areas (Figure 1). 5 cases of mitotically active cellular fibromas were retrieved along with seven cases of cellular fibromas. Mitotically active cellular fibromas had mean age of 46 years and mean size of 9.7 cm. Follow up of 37 cases was available including 3 cases each of mitotically active cellular fibroma and cellular fibroma. Only one case of mitotically active cellular fibroma showed recurrence, 14 months after initial surgery.

### Thecomas

Out of 26 cases of thecomas, one case behaved in aggressive fashion extending into the urinary bladder. Right ovary was involved in 11 cases and left ovary in 12 cases (n = 23) The tumor presented at stage I, in all cases except one in which tumor infiltrated the urinary bladder wall, and only in this case, moderate atypia and significant mitoses (3/10 HPF) were present. The slides of this unusual case were reviewed and no granulosa cell component was identified even with reticulin stain. Follow up of 13 cases was obtainable and all the patients were tumor free with mean follow up period of 94 months.

### Fibrothecomas

Total 47 cases of fibrothecomas were recovered out of which one was associated with endometrial hyperplasia and one was found with carcinoma cervix. Age range was 2 years to 80 years and median age was 50 years. Right side was more commonly involved in 28 cases and left in 12 cases. Two cases were bilateral. Follow up of 19 patients was available and all the patients were alive and healthy.

### Sertoli-leydig cell tumor

Total 34 cases of sertoli-leydig cell tumor (SLCT) were found. Proper surgical staging was done in 19 tumors, with 11 presenting at FIGO stage II or higher. Spectrum of these tumors according to differentiation and the information about tumor stage is given in Table 4. Detailed follow up of only 12 cases was available and 2 patients developed metastasis and died of disease after 9 and 23 months respectively despite Cisplatin based chemotherapy.

**Table 4:** Sertoli-leydig cell tumors; frequencies of various types

**Number of cases (percentage)**	**Differentiation**	**Surgical stage (FIGO)**	
		**Stage I**	**Stage II or higher**
3 (8.8%)	Well differentiated (Meyer Type I)	2	-
15 (44.1%)	Intermediate differentiated (Meyer Type II)	4	4
10 (29.4%)	Poorly differentiated (Meyer Type III)	1	4
4 (11.8%)	With heterologous elements (teratoid androblastoma)	-	2
1 (2.9%)	Retiform(“netlike”)	1	-
1 (2.9%)	Metastatic	-	1

### Sclerosing stromal tumor

26 cases of sclerosing stromal tumor (SST) were diagnosed and 13 involved left ovary. Ten arose in right ovary and information about side was missing in 3 cases. In all cases except one, capsule was intact. Follow up of only 17 patients was available and no recurrence or metastasis was seen with mean follow up period of 134 months.

### Steroid cell tumors

Steroid cell tumors (SCT) were total 10 in number. All of the tumors were stage-I at time of diagnosis. Additional Immunohistochemical stain used was Melan A and WT-1 were positive in all of cases when applied. Follow up of 8 patients was available and one tumor recurred after 21 months of surgery.

### Sex cord tumor with annular tubules

Sex cord stromal tumor with annular tubules was the diagnosis in 4 cases, with a wide age range of 15 to 57 years. 2 tumours having detailed clinical history had associated Peutz Jegher’s syndrome.

### Bilateral tumors

Out of four bilateral cases, two were fibrothecomas, one was AGCT and one was sertoli leydig cell tumor. No two different tumors occurred bilaterally at same time.

### Immunohistochemical analysis

Immunohistochemical studies were done in 241 patients and most common panel used included Cytokeratin AE1/AE3, Cytokeratin Cam 5.2, Calretinin, inhibin, WT1 and Anti-Smooth Muscle Actin (ASMA). EMA was also used (n = 121) and it was negative in all the cases, a very important differentiating point.

## Discussion

The SCSTs account for approximately 7% of all ovarian tumors [1,3,4]. Although these usually present at younger age group, there is wide age range of presentation and wider morphologic spectrum of these tumors [3,5]. The coexistence of two different types of sex-cord stromal tumors is also extremely rare [9].

In Pakistan, little research has been done focusing on epidemiology of ovarian sex cord stromal tumors. In this retrospective analysis, we aimed to report the experience with a large series of these rare tumors in a major tertiary care center in Pakistan.

Most of the characteristics of our study population were similar to those reported in the literature. The commonest symptoms are abdominal pain and distention along with menstrual disturbances. However in our study population, the hormonal changes were not very frequently observed. In our series, just under half of women were below the age of 50 at the time of diagnosis and fertility sparing surgery is often desired among these women. So the nature of specimen listed in Table 3 vary according to the tumor type hence reflecting the median age of patient at the time of diagnosis and the surgical impression of malignancy intra-operatively. Moreover there was little variation in size of tumors among the sub-groups, hence did not correlate significantly with the benign or malignant nature of tumor. In children, the most frequent tumors were JGCTs followed by SST, SLCT and fibrothecomas.

Newer entities were also encountered though less frequently as reported in literature to date. Total 5 mitotically active cellular fibromas were found, exclusive of 7 cellular fibromas.

GCTs are sex-cord-stromal tumors that make up 70% of all such tumors but in our study these tumors comprised of 43.5% of all the sex cord stromal tumors that were diagnosed during the study period. The granulosa cell tumors are divided into adult (95%) and juvenile (5%) types based on specific diagnostic histological findings; however our observation showed that juvenile granulosa cell tumors were around 10% of all GCT which is bit higher than reported in other large series. The JGCT occurred in premenarchal girls and young women [8-11].

Inhibin was positive in all the granulosa cell tumors. Cytokeratin’s dot positivity was observed in most of the cases of AGCT (76.5%). In JGCTs, Cytokeratin negativity was one of the major helpful features to rule our epithelial malignancy despite greater nuclear pleomorphism and mitotic activity than adult granulosa cell tumor. Molecular analysis is also helpful in diagnosis of AGCT, which was not available in our set-up. Complete follow-up data of only 86 patients was available with mean follow-up duration of 37 months. In our study population, late recurrences with the granulosa cell tumors were not infrequent, encountered in 5 patients, which re-emphasizes the need for long term follow-up of these patients [12-14]. When the disease could not be completely resected or recurred after surgical resection, prognosis was poorer with 1 patients dying soon after recurrence. Metastasis was observed at the time of presentation in 13 of the cases and subsequent metastasis was observed in 4 of patients.

Addition of Calretinin and WT-1 these two antibodies was found to be quite useful in the diagnosis of ovarian SCSTs in equivocal cases [12,14].

Among total 98 cases of fibromas, the mean age of patients was in accord with the international data i.e. 42.2 years. Median age was 52, which was just slightly lower than the international published data. The seven Cellular fibromas had mean size of 14.5 cm, which is larger than the previously reported studies. 5 cases of mitotically active cellular fibromas had mean age of 46 years and mean size of 9.7 cm, which is comparable to the past series [15,16]. One case of fibroma was associated with carcinoma cervix and interestingly one case each with carcinoma of endometrium and endometrial hyperplasia, once again proving that these tumors are hormonally active as were many thecomas because endometrial pathology was present in 11 out of 26 cases of thecomas. [16]. Calretinin proved to be more sensitive than inhibin. In addition, we tested few of our subset of fibromas with CD10, because ovarian endometrial stromal sarcomas may have areas that resemble fibroma [17]. Follow-up of 37 cases was available including 3 cases each of mitotically active cellular fibroma and cellular fibroma. No metastasis was observed complying to the benign nature of tumor, however one case of mitotically active cellular fibroma recurred, 14 months after surgery.

Most of the ovarian sex cord stromal tumors, in which information regarding the FIGO stage was available, presented at Stage I. FIGO staging is the most important, globally accepted, prognostic factor. Most of fibromas and thecomas presented at stage I, in all cases except one in which tumor infiltrated the urinary bladder wall, and only in this case, moderate atypia and significant mitoses (3/10 HPF) were present [18,19]. Most common specimen received was unilateral oophorectomy, relating to the clinically and radiologically benign nature of most of the tumors. Predominantly only in granulosa cell tumors and Sertoli Leydig cell tumors among our study population, surgical staging procedures were done. And this number was also very low, but owing to no nodal metastasis in these cases, this might be appropriate. In patients with SLCT where proper surgical staging procedure was done (n = 19), it was found that majority of tumors (n = 11) presented at higher stage i.e. FIGO stage IIA/IIB or higher. Although mostly these tumors present at stage I, the probable explanation for presenting at higher stage in our observation could be the very low number of well-differentiated SLCT.

Total 34 cases of sertoli-leydig cell tumor were found. Mean age 35.2 years. Mean size 15.4 cm. Poorly differentiated were a little more frequent as compared to previous study data but SL tumor with heterologous elements were somewhat less. [20-22].

SST is a very rare ovarian stromal tumour that was for the first time described by Chalvardjian and Scully. 26 cases of sclerosing stromal tumor were diagnosed with the mean age of 28.7 years, which was comparable to other studies [23-26]. However age range was slightly wider, 10 to 64 years. In all cases except one, capsule was intact. Analysis of cut surface showed that most of the cases had solid and cystic areas, however 4 cases had entirely solid surface. The spindly cells were stained strongly for both ASMA and desmin along with Inhibin and theca like component stained positive with CD 99 and calretinin. Metastatic signet-ring cells were excluded with the negative staining for keratin and EMA.

Steroid cell tumors were total 10 in number. Mean age was 45.2 years, which was in accord with the previously reported mean age for this tumor [20,21]. Most common presentation was virilizing symptoms. Additional Immunohistochemical stain used was Melan A, which was positive in all of cases when applied. WT-1 was also positive in all the cases. Cytokeratins were negative in all the cases.

Sex cord stromal tumor with annular tubules was the diagnosis in 4 cases, with a wide age range of 15 to 57 years. Two of the cases were found to be associated with of Peutz-Jeghers Syndrome. In the other two cases, adequate history was not available. Because of this missing data, the strong association between SCTAT and Peutz-Jeghers Syndrome could not be re-substantiated.

Out of four bilateral cases, two were fibrothecomas, one was AGCT and one was sertoli leydig cell tumor. No two different sex cord stromal tumors occurred bilaterally at same time, however 8 fibrothecoma group tumors and 12 granulosa cell tumors were found to be associated with contralateral ovarian cystadenofibroma.

To our knowledge, this is first kind of study of long duration and large data set, addressing ovarian sex cord stromal tumors in Pakistani population specifically. Also our set-up is one of the largest anatomical pathology and oncology setup with laboratory specimen collection centres all over Pakistan, so representation of all of the parts of country has been done in this study. Although referral/second opinion cases were also included in this study so unusual cases such as AGCT were more commonly observed rather than Fibroma/FT as in many previously done international studies. However in our opinion this might not have affected the results to a larger extent as the other unusual and complex tumor i.e. SLCTs were encountered much less frequently as opposed to international data.

One of the drawbacks and deficiency of our study was unavailability of long term follow up of about half of the patients, which might be required to see the exact recurrence rate and patterns of metastasis of ovarian sex cord stromal tumors in our population [27-30].

## Conclusions

The ovarian sex cord stromal tumors are rare ovarian neoplasms in Pakistani population. The relative frequencies and patterns of presentation are more or less similar to the international data, however Sertoli-Leydig cell tumors were less frequently encountered. As the morphology of sex cord stromal tumors vary widely, immunohistochemical studies are mandatory to exclude epithelial malignancies, as the treatment option for the two, vary widely.

In future, there is need for long term longitudinal studies to see the prognostic implications of various subtypes and stage of these tumors.

### Consent

Informed consent was taken from all the reported patients with follow up.

## Abbreviations

SCST: Sex-cord stromal tumors; EMA: Epithelial membrane antigen; CK: Cytokeratin; FIGO: International Federation of Gynecology and Obstetrics; AGCT: Adult granulosa cell tumor; JGCT: Juvenile granulosa cell tumors; SLCT: Sertoli-leydig cell tumor; SST: Sclerosing stromal tumor; SCT: Steroid cell tumors; ASMA: Anti-smooth muscle actin.

## Competing interests

All the authors declare that there are no competing interests.

## Authors’ contributions

SH, NK and RI devised the study and supervised the data collection. SH, NK, RI, SF and AM contributed to the discussions. SH and AZ collected the data and performed the analyses. All authors contributed to the discussions. All authors read and approved the final manuscript.
